# Moral identity test (MIT) for children: reliability and validity

**DOI:** 10.1186/s41155-019-0120-9

**Published:** 2019-02-28

**Authors:** Kerem Coskun, Cihan Kara

**Affiliations:** Department of Primary Education, Artvin Coruh University, Sehir Kampusu, 08000 Artvin, Turkey

**Keywords:** Moral identity, School children, Test development

## Abstract

**Background:**

The purpose of the present study is to develop the Moral Identity Test (MIT) which measures the moral identity of primary school children.

**Methods:**

The present study was designed as survey research and 516 primary school children were included in the sample. Data were analysed with corrected item-total correlation, exploratory factor analysis (EFA), internal consistency analysis, convergent validity analysis, and item response theory (IRT).

**Results:**

As a result of the data analysis, it was found that the MIT consists of one construct with ten items and its internal consistency coefficient is .93.

**Conclusions:**

It was concluded that the MIT can generate reliable and valid results in measuring the moral identity of primary school children whose ages vary between 7 and 11 years.

**Electronic supplementary material:**

The online version of this article (10.1186/s41155-019-0120-9) contains supplementary material, which is available to authorized users.

## Introduction

Moral development includes various concepts such as moral reasoning, moral socialisation, moral character, moral judgement, and moral action. Those concepts were developed to predict the coherency between moral judgement and moral action. Moral identity is one of those concepts. Moral identity is defined as self-regulation that motivates humans to moral action (Aquino & Reed, 2002; Blasi, 1984; Damon & Hart, 1992). According to Aquino and Reed (2002), moral identity is a kind of social identification that humans use to build up their self-definition. In accordance with this definition, the more children behave in accordance with parental expectations and rules, the more they perceive themselves as being a good person, are more likely to apologise for their wrongdoings and are concerned with others’ disobedience (Kochanska, 2002). Moral identity is also viewed as self-commitment which is consistent with the sense of self in lines of action. Consistency between self-commitment and action both contribute to the welfare of others and protect individuals from others. As a result, moral identity entails coherency between self-goals and actions (Atkins, Hart, & Donnelly, 2004). In other words, moral identity is the unity between self-systems and morality (Colby & Damon, 1992).

Moral identity is an important source of moral motivation (Hardy & Carlo, 2011). Damon and Hart (1992) argue that moral identity is the best predictor of moral actions and commitments. Blasi (1993) argued that moral judgement can reliably predict moral behaviour when moral judgements are filtered through responsibility judgements and lead to action via the inclination for self-consistency. As result, less difference between sense of morality and personal goals distinguishes highly moral people from others (Colby & Damon, 1992).

According to social cognitive theory, moral identity is a complex structure related to moral values, goals, traits, and behavioural scripts (Aquino & Reed, 2002; Lapsley & Narvaez, 2004). Knowledge structure is a result of learning. Learning requires life experiences (Aquino & Reed, 2002; Blasi, 2004; Lapsley & Narvaez, 2004). Feeling an obligation to participate in moral actions is associated with moral identity based on the desire to maintain self-consistency (Aquino, Freeman, Reed II, Lim, & Felps, 2009; Blasi, 1980; Blasi, 1993). An individual possessing moral identity feels obliged to behave in accordance with behavioural prescription so as to avoid self-condemnation. However, those whose moral identity is less central to self-concept are not motivated to become involved in moral actions (Aquino et al., 2009). According to the social-cognitive perspective, self-concept is a network of identity schemas. The influence of any identity encompassing the working self-concept allows access to moral schemas and behavioural scripts in any given situation. When moral identity is accessible, a person describing themselves as a moral individual generates moral action. Therefore, situation and environmental factors influence the action of moral schemas and self-concept, and accessibility of moral schema.

Schemas play key roles in instilling healthy moral identity in children. Schemas are the mental construction and representation related to an object and event in the mind. Based on this definition, it can be concluded that moral schema is a mental representation about what moral actions are and what it means to be a moral person (Hardy & Carlo, 2011; Lapsley & Narvaez, 2004). Schema appears and develops in interpersonal relationships and by observing others in social environments. Therefore, schema is formed through social interactions and open to change. Moral schema emerges in social interactions. Social interactions, however, build up self and identity because responses to social environment lead to feedback related to the response of the individual. This feedback, in turn, constructs self and identity. In turn, as a result of social interaction, mental prototypes about how to behave and what is moral develop in the individual. Therefore, moral schemas are necessary and must be readily accessible for social information processing in order to construct moral identity (Narvaez, Lapsley, Hagele, & Lasky, 2006). Moral identity requires a deep network of cognitively accessible mental schemas (Lapsley & Narvaez, 2004).

Social experiences such as helping, caring, and sharing provide scripts or event representations about moral actions (Hardy & Carlo, 2011). Social experiences through either vicarious experience or direct experience build up behavioural scripts in memory that allow those behaviours to become more automatic and self-evaluative. This behaviour links moral affect to related schemas. This moral schema leads the individual to perform or avoid certain behaviour (Reimer, 2003).

Moral identity consists of three components of will power, moral desire, and integrity. Willpower is a motivation to cope with external and internal impediments in pursuing a long-term aim. Integrity allows integration of commitment to sense of self. Moral desires manage willpower and integrity, providing a framework in which action is significantly moral (Blasi, 2004).

### Rationale of the study

Moral identity plays a crucial role in predicting and explaining moral action that it was defined in different ways and various models were developed for it. Blasi (2004) developed a self-model that handled moral identity with moral judgement. The model developed by Atkins et al. (2004) suggests that moral identity arises from moral judgement, self-understanding, and social opportunities. There are a plethora of definitions about moral identity (Aquino & Reed, 2002; Atkins et al., 2004; Blasi, 1993; Damon & Hart, 1992; Hardy & Carlo, 2011), and there is a large body of study which explains moral identity in an operational way (Aquino & Reed, 2002; Aquino et al., 2009; Frimer & Walker, 2009; Hardy & Carlo, 2005; Hart, 2005; Lapsley & Narvaez, 2004; Pagano, 1991; Reynolds & Ceranic, 2007; Splitter, 2017). However, there are very few studies which developed a self-report instrument for moral identity (Aquino & Reed, 2002) and there is no scale which assesses the moral identity of primary school children through performance tests. Therefore, the present study aims to develop a test which assesses the moral identity of primary school children. Moral identity among adults and adolescents has been studied, whereas there is no research which seeks to investigate moral identity among children who attend primary school (Damon & Gregory, 1997; Hart, Atkins, & Ford, 1998; Henry, 1987; Splitter, 2017; Youniss & Yates, 1997).

Learning related to moral behaviour has become crucial in school environments. Because not only are schools the places where cognitive abilities of students are improved, but also they are responsible to teach moral behaviours. Moral identity of primary school children can be observed, measured, and monitored through moral identity. Consequently, a moral identity scale can be said to help teachers monitor moral behaviour of primary school children and evaluate classroom activities of learning outcomes for moral learning outcomes.

## Method

### Design of the study

The present study was designed as survey research, one of the quantitative research traditions, due to the fact that the present study aims to generalise research findings over a population and testing aims to assign scores and numbers to behavioural dispositions and characteristics (Fraenkel, Wallen, & Hyun, 2012; Murphy & Davidshofer, 2005). The purpose of the study is to develop a performance test which yields reliable and valid results in measuring moral identity among primary school children. Test development includes two phases of construction of the test and norming and standardising the test (Murphy & Davidshofer, 2005).

### Construction of the test

Test construction involves item writing, item content, and item response.

#### Item writing

Moral rules, moral behaviours, and moral principles are taken for granted in the socio-cultural environment. Therefore, semi-structured interviews were conducted with a primary school teacher and a parent to ruminate on and understand what a typical Turkish primary school child experiences and how he behaves during events which require moral judgement and moral action in home and school settings. In the semi-structured interview, the primary school teacher was asked to give examples of moral behaviour and immoral behaviour among their students. The same questions were asked to the parent. Statements of the teacher and the parent functioned as a basis for situations in which primary school children conduct hypothetical thinking. A literature review was carried out in order to give items a theoretical background. For this reason, Atkins et al. (2004), Blasi (1984, 2004), Colby & Damon (1992), Hardy & Carlo (2011), Kleinberger, (1982), and Lapsley & Narvaez (2004), were reviewed. The literature review and statements of the primary school children and the parent enabled integration of fact and theory. As a result of the interviews and the literature review, 18 items were written.

#### Item content, item response alternatives, and scale construction

Blasi (2004) argued that moral identity requires three components of willpower, moral desire, and integrity. In accordance with Blasi (2004)’s argument, it was decided that item responses would have three alternatives of “I warn”, “I abstain”, and “I don’t care”. Response of “I warn” to the items proves the existence of willpower, moral desire, and integrity. Response of “I abstain” to the items indicates the existence of moral desire but discloses a lack of willpower and integrity. Response of “I do not care” to the items is an indicator of deficiency of willpower, moral desire, and integrity. Therefore, the response of “I warn” was given 3 points, the response of “I abstain” was scored with 2 points, and the response of “I do not care” was given 1 point. As a result of the objective construction of response alternatives, the MIT has maximal performance (Cronbach, 1970). In addition, it is known that children find drawings interesting so they make an assessment of children’s skills easy (Lewis & Greene, 1983). Therefore, it was decided that drawings which depict facial expressions related to “I warn”, “I abstain”, “I do not care” was added to the response alternatives of the Moral Identity Test. As a result, response alternatives involve written expressions and visual expressions and written and visual stimuli were integrated for each of the 18 items. After integration of written and visual stimuli for response alternatives, test-construction was finalised and a draft of the MIT was created.

Determining clarity in expression and theoretical relevance of items with phenomenon which is observed and measured is a key step in test development. Determining clarity in the expression of the items requires an expert of Turkish language while examination of the 18 items in terms of entails expert of primary education and moral education. Therefore, the draft MIT was sent to five experts whose expertise is related to moral development, primary teaching, and Turkish language to determine whether it could assess the skills targeted, includes necessary elements of moral life, and has good clarity in Turkish. Two of them are expert on moral education, two of them are expert on Turkish language, and one of them is expert on primary education. After feedback was received from the experts, necessary corrections were made and the final draft of the MIT was created.

#### Norming and standardising the test

Norming and standardising the test involves three stages of norming and defining the population, selection of sample, and standardisation (Murphy & Davidshofer, 2005).

#### Norming and defining the population

The basic purpose of norming is to determine the normative group. The intended use of the test determines the normative group (Murphy & Davidshofer, 2005). The Moral Identity Test (MIT) aims to assess the moral identity of primary school children whose ages range between 7 and 10 years. Therefore, the population of the study includes primary school children (Additional file 1).

#### Selection of the sample

Determining the target population is followed by selection of the sample. Due to financial and time constraints, sampling strategies were used to derive a representative sample from the population (Murphy & Davishofer, 2005). Cluster random sampling strategy was employed owing to the impossibility of listing all primary school children and lack of inclusion of age groups in the sample the same as the population (Fraenkel et al., 2012). Before data collection, official and ethical approval was taken from Provincial Directorate of National Education in Artvin, which is in northeast part of Turkey.

Cluster sampling strategy was used to collect data due to the impossibility of random sampling, administrative difficulties, and listing all members in the population. Grades in primary school years were determined as reference unit. Moreover, Turkey is such a large country that it is difficult to reach primary schools from all regions of it. Participants studying primary school grades were chosen. As a result of cluster sampling, 516 primary school children were recruited. Of them, 116 were included in validation studies and 400 of them were recruited for exploratory factor analysis (EFA), item analysis, reliability analysis, and item response theory (IRT). One hundred eighty-nine (47.2%) of them are female, and 211 (52.8%) of them are male primary school children. As for distribution of the participant children to the age groups, 100 primary school children fell into each of the age groups. The mean age of the sample was found as 8.5 and its standard deviation was observed as 1.11.

#### Standardisation

Standardisation is the last stage in test development. The major aim of standardisation is to remove extraneous variables, which could influence test performance of the participating children. In order to standardise the testing process, testing conditions and procedures must be kept as invariant as possible in every administration of the test (Murphy & Davidshofer, 2005).

Schools were visited, and the aim of the study was explained to primary school children and their teachers. After they accepted voluntary participation in the study, a colour printed version of the MIT was given to them. The MIT, its items, and response alternatives were explained to the participant primary school children. They were asked to follow instructions from the researchers, not to pass to the next items without instruction from the researchers, not to speak their responses so that they could think about the items and their responses, and avoid influencing each other or were influenced by any peers. As a result, testing conditions and procedures were kept as stable as possible. Overall, the participant children completed the test within 20 min.

## Results

### Data analysis

Data analysis includes three main strategies of disclose latent trait variance, structural analysis, and item response theory (Clark & Watson, 1995; Loevinger, 1957). Structural analysis and item response theory were used to construct the MIT. Structural analysis was conducted through SPSS, and item response theory analysis was carried out via STATA.

### Structural analysis

Item-total correlations, internal consistency, exploratory factor analysis, validation, and item response theory (IRT) were used to reveal structural characteristics of the MIT. This stage plays a key role because the MIT measures moral identity among primary school children through theoretical-based measurements (Clark & Watson, 1995). Therefore, item analysis through corrected item-total correlation and item response were conducted to determine internal consistency and unidimensionality of the MIT.

### Item-analysis with item-total correlation

Item analysis through corrected-item total correlation helps to identify the items that do not correlate with the overall test and measure different dispositions or traits. Corrected item-total correlation analysis indicated that all of the items are correlated with the overall test score. In other words, there is no item whose corrected item-total correlation is lower than .30 (Nunnally & Bernstein, 1994). Therefore, it was decided to include 18 items in exploratory factor analysis. Results of item analysis are shown in Table 1.

**Table 1:** Item analysis results

Item	Corrected item-total correlation
Item 1	.51
Item 2	.60
Item 3	.58
Item 4	.74
Item 5	.45
Item 6	.48
Item 7	.69
Item 8	.38
Item 9	.64
Item 10	.65
Item 11	.79

#### Exploratory factor analysis

Exploratory factor analysis (EFA) allows identification of inter-correlated items and clusters them under the same constructs (Field, 2009). Before EFA, the Kaiser-Meyer-Olkin (KMO) coefficient and Barlett’s test were examined. KMO was found as .92 and Barlett’s test was observed to be significant (*χ*^2^ = 3117, 045; *p* ≤ 0.001). Based on the results of KMO and Barlett’s test, the sample was found to be large enough to conduct EFA (Field, 2009; Henson & Roberts, 2006). Varimax rotation method was used because of the fact that varimax rotation makes factors more interpretable as clusters and disperses factor loadings. Eigenvalue was regarded as reference to decide the number of factors. EFA was conducted and it was observed that there were three factors whose eigenvalue is over 1.00. However, it was found that item 1, item 3, item 5, item 6, item 8, item 10, item 14, and item 16 were included in two factors due to the difference of less than 1.00 in factor loadings. These eight items were discarded from EFA while item 2, item 4, item 7, item 9, item 11, item 12, item 13, item 15, item 17, and item 18 were kept in EFA. EFA was carried out again and it was found that there is one factor whose eigenvalue is over 1.00. One factor solution with ten items explains 64% of total variance. The number of factors must explain at least 50% of the total variance (Merenda, 1997). As a result, it was concluded that one-factor solution with ten items can identify a strong construct from the data. It was also seen that factor loadings of the items vary between .634 and .871.

#### Internal consistency

Internal consistency is a reliability method that reveals the precision of test (Briggs & Cheek, 1986; Cronbach, 1951; Murphy & Davidshofer, 2005; Nunnally & Bernstein, 1994). The Cronbach’s alpha coefficient indicates the reliability coefficient based on internal consistency. As a result of the analysis, the internal consistency coefficient of Cronbach alpha was found to be .93. EFA results and internal consistency analysis results are displayed in Table 2.

**Table 2:** Exploratory factor analysis results

Item	Factor loadings	M	SD	Alpha if item deleted
Item 2	.634	2.27	.91	.93
Item 4	.839	2,26	.90	.92
Item 7	.820	2,27	.93	.92
Item 9	.728	2,27	.87	.92
Item 11	.871	2,26	.91	.91
Item 12	.705	2,21	.92	.93
Item 13	.882	2,24	.83	.91
Item 15	.794	2,22	.88	.92
Item 17	.835	2,56	.70	.92
Item 18	.726	2,14	.82	.92

Eigenvalues = 6.19 total variance explained; 64% KMO = .92 Barlett’s test; *χ*^2^ = 3117, 045; *p* ≤ 0.001

### Construct validity

Construct validity reveals whether test scores measure a specific construct (Campbell & Fiske, 1959; Murphy & Davidshofer, 2005). In order to test construct validity, a subtype of construct validity called convergent validity was examined. Convergent validity is a parameter which indicates the degree to which two measurements of constructs are related to each other. Emotional intelligence and moral identity are affective dimensions of personality. Development in one measure supports the other measurement. In other words, it can be theoretically claimed that children with high emotional intelligence score better in the MIT than children with lower emotional intelligence. Ten Years Emotional Intelligence (TYEIS) developed by Coskun, Öksüz, and Yılmaz (2017) and the MIT were administered to 116 primary school children who are 10 years old in order to test the construct validity through the correlation between the two measurements.

The correlation between TYEIS and MIT was found to be .46. Findings in Table 3 indicate that there is positive, moderate, and significant correlation between the two measurements (*r* = .46; *p* < 01). Based on the correlation analysis, the MIT has good convergent validity.

**Table 3:** Results of correlation between the TYEIS and the MIT

Measurements	*N*	*r*	*p*
The TYEIS	116	.46*	.00**
The MIT			

*Two-tailed

**p < .01

### Precision of MIT through item response theory (IRT)

Item analysis based on a classical test discloses a great deal about the items. However, item analysis based on classical test development cannot reveal factors that are related to test takers and influence test performance. Item response theory (IRT) is handy to find nonlinear relationships between the test takers’ characteristics and their responses to a test item because of the fact that IRT enables computation of a standard error of measurement for latent traits (Edelen & Reeve, 2007; Hambleton & Swaminathan, 1985; Murphy & Davidshofer, 2005).

#### Item characteristic curve (ICC)

The item characteristic curve is an essential function of the IRT. Therefore, construct analysis of the IRT is carried out based on the ICC. The ICC indicates the chances of responding with a correct answer through attributes or traits measured by a test. In other words, the ICC provides information about what extent the attribute measured by a test should be possessed by the individual in order to correctly respond to the item. The ICC also presents information about the item’s difficulty, power of discrimination, and the probability of responding correctly by guessing (Murphy & Davidshofer, 2005).

**Fig. 1:**
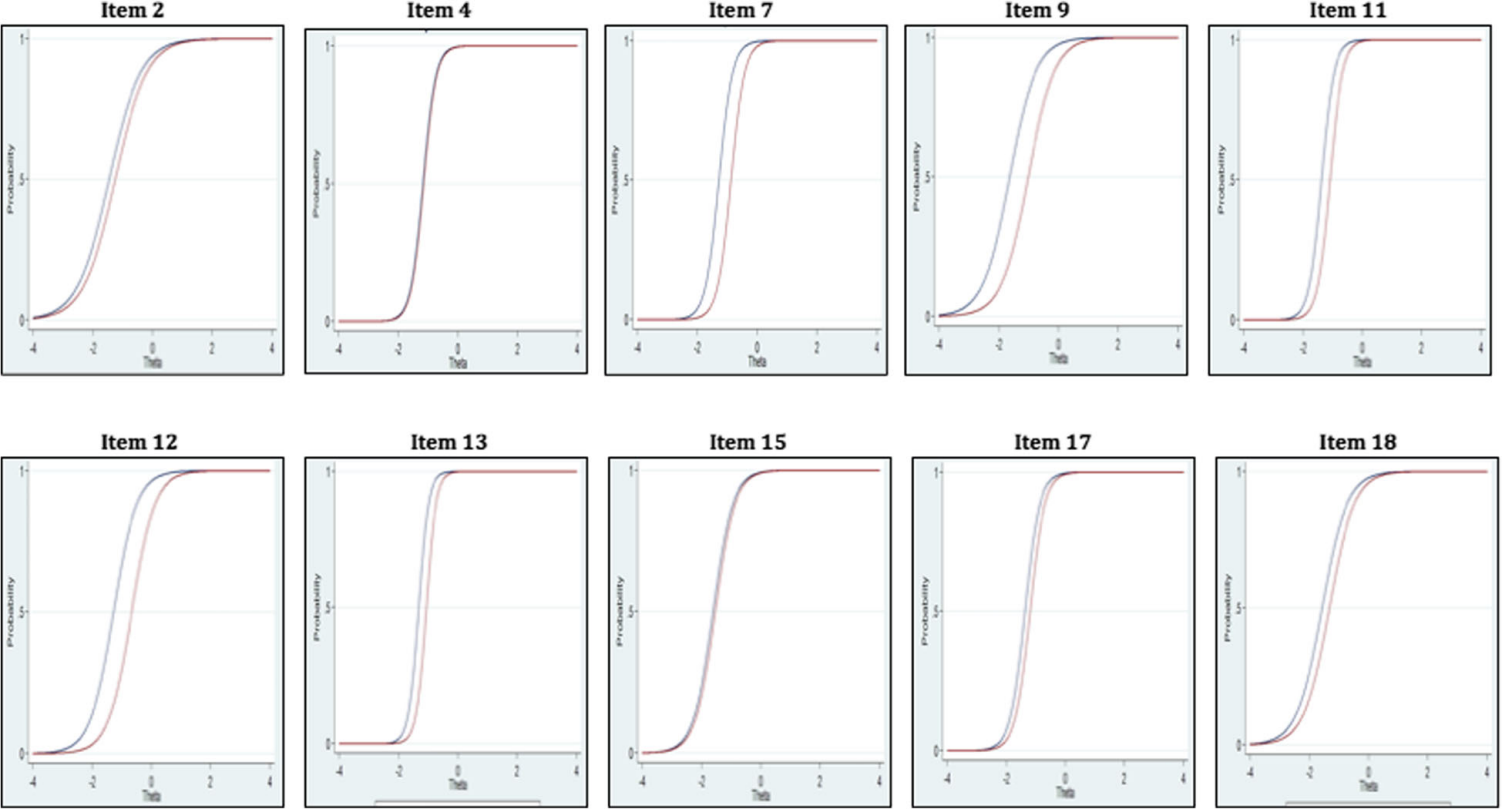
ICC curves

The ICC analysis was conducted with the graded response model (GRM) due to the fact the MIT has three graded response alternatives and item responses are not dichotomous (Samejima, 1969). Results of the IRT related to the ICC are shown in Fig. 1 and Table 4.

**Table 4:** GRM item parameter estimates

Item	*a*	*b*_*1*_	*b*_*2*_
Item 2	1.87	− 1.46	− 1.26
Item 4	4.80	− 1.18	− 1.13
Item 7	4.43	− 1.27	−.87
Item 9	2.24	− 1.64	− 1.02
Item 11	4.72	− 1.38	− 1.09
Item 12	2.53	− 1.28	−.66
Item 13	5.98	− 1.32	− 1.07
Item 15	2.90	− 1.61	− 1.54
Item 17	4.00	− 1.37	− 1.20
Item 18	2.35	− 1.57	− 1.32

The value of *a* indicates the power of the discrimination between better performers and poor performers (Hambleton & Swaminathan, 1985). Results of the GRM revealed that all of the items in the model have over 1.00 *a* value. Hence, it was concluded that all the items can discriminate between better performers and poor performers. *B* values present information about the difficulty of the items and location of item parameters where the participant primary school children have .50 chance of responding correctly to the items. *B* values vary between −.1.64 and −.66. Consequently, findings of Table 1 revealed that items of the MIT are capable of discriminating better performers from poor performers and possessing adequate item difficulty.

## Discussion

The results of the study indicate that the MIT can yield reliable and valid results in measuring the moral identity of primary school children whose ages range between 7 and 11 years. Based on analysis results, it was also observed that it includes one-factor solution with ten items. The internal consistency coefficient of the MIT was found to be .93. In addition, all of the items in the MIT have over 1.00 *a* value; hence, it can discriminate well between primary school children with lower moral identity and children with higher moral identity.

Although moral identity has been studied a great deal in the relevant literature, there is only one scale about moral identity which is for adults and was developed by Aquino and Reed (2002). As a result of the study, the MIT was developed to measure the moral identity of primary school children. While the moral identity scale developed by Aquino and Reed (2002) is a self-report scale, the MIT is a test of maximal performance due to the fact that its response alternatives were graded in accordance with the moral identity model constructed by Blasi (1984). This characteristic makes the MIT theoretically oriented.

Moral identity refers to coherency between sense of self and action (Atkins et al., 2004; Colby & Damon, 1992). As understood from the description, moral identity plays a key role in adjustment to social environments. Adjustment to social environment is a major aim of the formal education process. Therefore, schools have a responsibility to teach moral skills to students, along with families. Schools foster moral identity by offering opportunities for enactment of moral behaviours, a moral environment which is similar to real-life conditions, and direct instruction through both extra-curricular activities and curricular activities (Atkins et al., 2004). Measurement and evaluation are essential parts of the instructional process to identify the impact of curricular activities. As for moral identity, measurement and evaluation provide feedback to teachers about what they have achieved and determine the impact of curricular activities. The MIT can also be used to monitor the moral development of students.

Development of moral identity is influenced by several factors. These factors can be classified as individual factors and contextual factors. Personality, attitudes, and cognitive development are considered as the individual factors. For instance, more cognitive development triggers more accurate moral information processing. More accurate moral information processing, in turn, makes children more adept in terms of moral identity. Family environment, social structure, institutions, and community organisations are the contextual factors. For example, a healthy and supportive family environment enables the development and integration of morals and identity (Hardy, 2008). The MIT can be used to clearly identify the influence of both the contextual factors and the individual factors on moral identity among primary school children.

Schemas, self-concept, self-understanding, and sense of self comprise moral identity. They are also influenced by cultural factors. The MIT was applied to Turkish primary school children. Their moral schemas, self-concepts, self-understandings, and senses of self were shaped by Turkish culture. Therefore, the MIT is culturally specific. The MIT can be adapted to different cultural contexts to extend its validity.

## Conclusion

The results of the study prove that the MIT produces valid and reliable results in assessing moral identity among primary school children and discriminates children with better response to the MIT from poor responders. The MIT is a maximal performance test so the test items and their responses, right and wrong answers, were determined based on the moral identity model developed by Blasi (1984). The EFA results indicated that item 1, item 3, item 5, item 6, item 8, item 10, item 14, and item 16 should be discarded from the rest of the analysis because those items were included in two factors. Item 2, item 4, item 7, item 9, item 11, item 12, item 13, item 15, item 17, and item 19 were kept in the test. The construct consisting of item 2, item 4, item 7, item 9, item 11, and item 12, item 13, item 15, item 17, and item 19 has a one-factor solution and is unidimensional. Convergent validity was tested that by applying the TYEIS and the MIT to 116 primary school children and medium, significant and positive correlation was observed between the two measurements. The MIT’s internal consistency coefficient was found to be .93. Therefore, the construct was analysed through item response theory (IRT), one of the modern testing traditions. The IRT results indicated that all the items in the MIT discriminate well between better responders and poor responders.

The MIT can be used in the evaluation of the impact of instructional activities about socio-emotional aspects of learning. The MIT can also be used by classroom teachers to observe and monitor the moral development of primary school children. The MIT can also be employed to determine the impact of individual factors and contextual factors on moral identity development among primary school children.

### Limitations of the study and implications for future research

In future research, the MIT could produce valid and reliable results in assessing the impact of the socio-economic status of primary school children on moral identity during the primary school years. The limitation of the study is that the MIT was designed and developed in terms of Turkish culture and was administered to Turkish primary school children. The MIT needs adaptation to different cultural environments in future research. On the other hand, test-retest reliability of the MIT has not been calculated due to the omission of the participant primary school children. In the future research, test-retest reliability of the MIT can be examined.

Moral identity is closely related to cultural and socio-economic variables. Investigation of correlation between moral identity cultural and socio-economic variables is beyond the present research. Future research can address to reveal the variation of moral identity according to the socio-economic status of primary school children and cross-cultural research can compare moral identity across different countries.

## Additional file

Additional file 1: Moral identity test: its English translation. (DOCX 120 kb)

## Abbreviations

1-MIT: Moral Identity Test
