# Supplementary figures and images for: Moral identity test (MIT) for children: reliability and validity
Source: Psicol Reflex Crit. 2019 Feb 28;32:7. doi: 10.1186/s41155-019-0120-9 (PMC6967304; doi:10.1186/s41155-019-0120-9)

**Additional file 1:** MORAL IDENTITY TEST: ITS ENGLISH TRANSLATION.


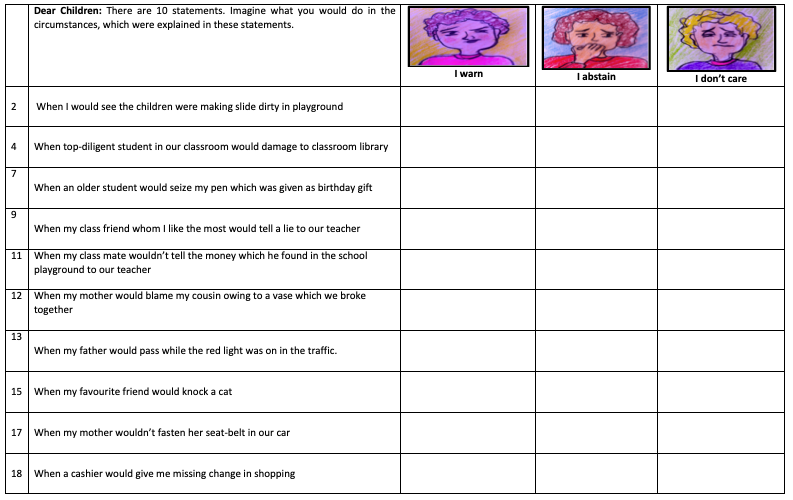

Supplement: Supplementary file 1 — Moral identity test: its English translation. (DOCX 120 kb) [file 41155_2019_120_MOESM1_ESM.docx]
